# Retrospective study of COVID-19 experiences in elite multinational aquatic athletes

**DOI:** 10.1038/s41598-023-40821-2

**Published:** 2023-08-26

**Authors:** Vencel Juhász, Emese Csulak, Liliána Szabó, Zsófia Ocsovszky, Dorottya Balla, György Nagy, Alessandro Zorzi, Andy I. M. Hoepelman, Béla Merkely, Hajnalka Vágó, Nóra Sydó, World Aquatics, Sports Medicine Committee, Cees-Rein van den Hoogenband, David Gerrard, Kevin Boyd, Christer Magnusson, Béla Merkely, Jim Miller, Farhad Moradi Shahpar, Edgar Ortiz, Josip Varvodic, Xinzhai Wang, Mohamed Yahia Cherif, World Aquatics, COVID-19 Task Force, Mohamed Diop, David Gerrard, Cees-Rein van den Hoogenband

**Affiliations:** 1 Heart and Vascular Center, Semmelweis University, 68 Városmajor Street, Budapest, 1122 Hungary; 2 Department of Sports Medicine, Semmelweis University, Budapest, 1122 Hungary; 3 University of Padova, Padua, Italy; 4 World Aquatics, Lausanne, Switzerland

**Keywords:** Epidemiology, Viral infection

## Abstract

This study assessed the experiences of elite aquatic athletes with coronavirus disease 2019 (COVID-19) during the first World Championship conducted without social distancing and an isolation “bubble”. An online questionnaire was completed by 812 athletes (22.7 ± 5.9 years, 467 females) to provide data on demographics, sports activity, severe acute respiratory syndrome coronavirus 2 (SARS-CoV-2) infection rates, symptoms, reinfection, vaccination status, and psychological aspects. The answers revealed that 49.4% of athletes had experienced SARS-CoV-2 infection. The infection rates varied significantly across different aquatic sports, with open water swimmers having the lowest (28%) and water polo players (67%) and artistic swimmers (61%) having the highest infection rates (p < 0.0001). The majority reported mild (51%) or moderate (27%) symptoms, while 16% remained asymptomatic. Reinfection occurred in 13%, and 10% of initial infections led to long COVID, with fatigue (65%) and shortness of breath (48%) being the most common long-term symptoms. Significantly, 92% of athletes received at least two vaccine doses and reported a positive vaccination experience (median score of 8 out of 10 for each shot). Mood changes and subjective performance drops significantly correlated with the overall experience scores (rho: 0.617, p < 0.0001, and rho: 0.466, p < 0.0001, respectively). In conclusion, most athletes experienced a benign disease course despite a relatively high infection rate. This study provides valuable insights into the COVID-19 experiences of elite aquatic athletes. The findings emphasize the importance of vaccination initiatives, monitoring psychological well-being and the need to fortify athletes’ resilience in the face of future health challenges.

## Introduction

The recurring Coronavirus Disease 2019 (COVID-19) waves have changed the world of sports in terms of specific regulations and concerns for athlete health. Evaluating the global impact of the COVID-19 pandemic on athletes is challenging, given the international variability in incidence and disease intensity^1^.

The first wave of the COVID-19 pandemic carried the risk of unforeseen health issues, which raised the question of the optimal return to play and potential cardiac and respiratory complications^2–4^.

However, the most significant questions regarding possible cardiac involvement have been answered^5,6^.

In addition, athletic performance may be influenced by the long COVID syndrome, which may lead to a delayed return to play^5,7,8^. In contrast with the initial extensive post-COVID screening practice^2,9,10^, current recommendations do not require comprehensive return-to-play examinations for all athletes^11,12^, as nowadays, the course of SARS-CoV-2 (severe acute respiratory syndrome coronavirus 2) infection is predominantly mild, without complications^13–16^.

Athletes participating in water sports have been particularly affected by the consequences of SARS-CoV-2 infection and long COVID syndrome. This has involved the postponement or cancellation of numerous international events, including the 2020 Tokyo Olympics^17^. However, the experience gained from the postponed Tokyo Olympics informed the organization of other significant sports events, including the 2022 FIFA World Cup^18^.

In addition, training opportunities were significantly limited due to the temporary closure of training sites, including swimming pools^17^. These factors altered periodised training programmes, affecting athletes’ physical performance and mental health^19,20^.

Fortunately, the development of vaccines has significantly reduced the number of severe cases^21,22^. Some concerns have arisen regarding vaccination-related myocarditis, and vigilant monitoring seemed rational, considering myocarditis’ implication in athletes^23^. Nevertheless, one must also consider optimal timing for vaccination to mitigate potential side effects and the impact on athletic performance^24^.

Currently, there is a dearth of data regarding aquatic athlete experiences during the pandemic. Athlete infection and reinfection rates and disease course relative to the general population are unknown, as are their vaccination status and ability to cope with added psychological hardship. In addition, most surveys and studies focus on local trends, with little application to global experiences and disease patterns.

This study aimed to assess the COVID-19 experience of a unique multinational cohort of elite aquatic athletes competing in the first global sports event without the imposition of an isolation “bubble”. We used a digital questionnaire to collect data on the infection rate, disease course, symptoms of SARS-CoV-2 infection, vaccination experiences and psychological well-being.

## Methods

As a part of a collaboration between the World Aquatics (AQUA—formerly Fédération Internationale de Natation (FINA)) Sports Medicine Committee, the AQUA COVID-19 Task Force and the Semmelweis University, we created an anonymised questionnaire focusing on the basic demographics, sports activity, previous SARS-CoV-2 infection(s), vaccination status and psychological aspects of the pandemic. This study presents cross-sectional, retrospective data. Hereafter, we use the term “COVID-19” to refer to the disease with clinical symptoms. Correspondingly, we employed the term “SARS-CoV-2 infection” to encompass both symptomatic and asymptomatic presentation.

### Questionnaire

Accredited athletes voluntarily completed the questionnaire during the 19th FINA 2022 World Championship in Budapest, Hungary, between June 13 and July 13, 2022, adding 10 extra days after the end of the event. The link and QR code pertaining to the form were distributed by volunteers during the event and were forwarded to the teams. The athletes filled in the English-language forms on their own electronic devices, usually taking 5–10 min to complete.

The questionnaire comprised 60 single-choice, multiple-choice, scale-type (1–10 points) and open questions. Firstly, we assessed the sociodemographic factors such as age, sex, nationality, aquatic discipline (swimming, water polo, artistic swimming, diving, open water swimming), training hours and years spent in training. We asked about previous SARS-CoV-2 infection(s), including their course and severity. Athletes were asked to identify the time of their infection(s) within six-month periods, starting with the end of 2019, the first half of 2020, and so on. Furthermore, we explored athlete vaccination status and collected psychological data concerning individual perceptions of the pandemic. The questionnaire used for data collection is found in Supplement 1. This study did not involve any SARS-CoV-2 screening or test results from the event. We excluded answer sheets with significant incompleteness or apparently contradictory information.

Athletes were categorised according to symptoms. Mild symptoms involved smell or taste disturbance, cough, fever, headache, fatigue (or significant weakness), palpitations (sensation of fast heart rate), eye pain, muscle or joint pain, and rhinorrhoea. Shortness of breath, chest pain and loss of consciousness without hospitalisation were regarded as moderate symptoms. Those who needed hospital care were identified as participants with a severe disease course, regardless of symptoms and symptom count. Symptom severity categories were created as follows: asymptomatic (1), mild (2), moderate (3), and severe (4). Based on the answers, we created an ordinal variable with seven categories regarding symptom length [no symptoms (1), maximum 24 h (2), 1–3 days (3), 4–6 days (4), 1–2 weeks (5), 3–4 weeks (6), beyond 4 weeks (7). Missed training time intervals had six different categories: no days (1), 1–3 days (2), 4–6 days (3), 1–2 weeks (4), 3–4 weeks (5) or more than 4 weeks of missed training (6). Symptoms that persisted beyond 4 weeks after the start of the infection fell into the category of long COVID symptoms^25^.

Mandatory quarantine duration was not uniform in time or location, and home training programmes might have been applied. For this reason, we decided to use only one pertinent variable of missed training time (Table 2) to identify those who kept training during quarantine and also those who had to extend training cessation beyond quarantine time for any reason. We excluded answer sheets containing contradicting information.

The athletes reported overall well-being during the pandemic from 1 to 10. A higher mark indicated a worse experience (1 point indicates the athlete experienced no effect from the pandemic, while 10 points reflects a terrible experience). Other psychologically oriented questions included rating mood changes, subjective performance drop and psychological support.

All methods complied with relevant guidelines and regulations. Informed consent was obtained from all participants and/or their legal guardian(s). Participants included in the calculations consented to anonymous data usage as part of the questionnaire. All experimental protocols and ethical approval were approved by the National Public Health Center (5200-6/2020/EÜIG) under the ethical standards laid out in the 1964 Declaration of Helsinki and its later amendments. No use of artificial intelligence was employed in this study.

Participants or the public were not involved in the design, conduct, reporting, or planned dissemination of the research findings.

### Statistical analysis and data management

Answers were analysed using descriptive statistics methods with MedCalc v.20.112 software. A p-value less than < 0.05 was considered significant. The Shapiro–Wilk test for normality was used. Continuous variables are shown as mean ± standard deviation or median with interquartile range as appropriate. Comparison between two independent groups was performed by t-tests or the Mann–Whitney test. Chi-squared and Fisher’s exact tests were used to study non-random associations between two or more categorical variables. Correlation between non-normal distribution variables was analysed using the Spearman rank correlation. Logistic regression was applied to predict dichotomous outcomes.

## Results

### Participants

Of the 907 responders, 92 did not permit data usage and were excluded from the calculations, and 3 answer sheets were not included in the calculations due to apparent inconsistencies. The final cohort comprised 812 athletes (39% of all World Championship competitors) with a mean age of 22.7 ± 5.9 years, of whom 57.5% were recorded as female. Swimmers (n = 333, 41%) were the most prevalent respondents across the aquatics disciplines, followed by artistic swimmers (n = 162, 20%), water polo players (n = 133, 16.4%), divers (n = 124, 15.3%), and open water swimmers (n = 60, 7.4%). The average weekly training time was more than 20 h (22.7 ± 10.1 h) with 14.6 ± 5.8 years of training history.

### SARS-CoV-2 infection incidence

**Figure 1:**
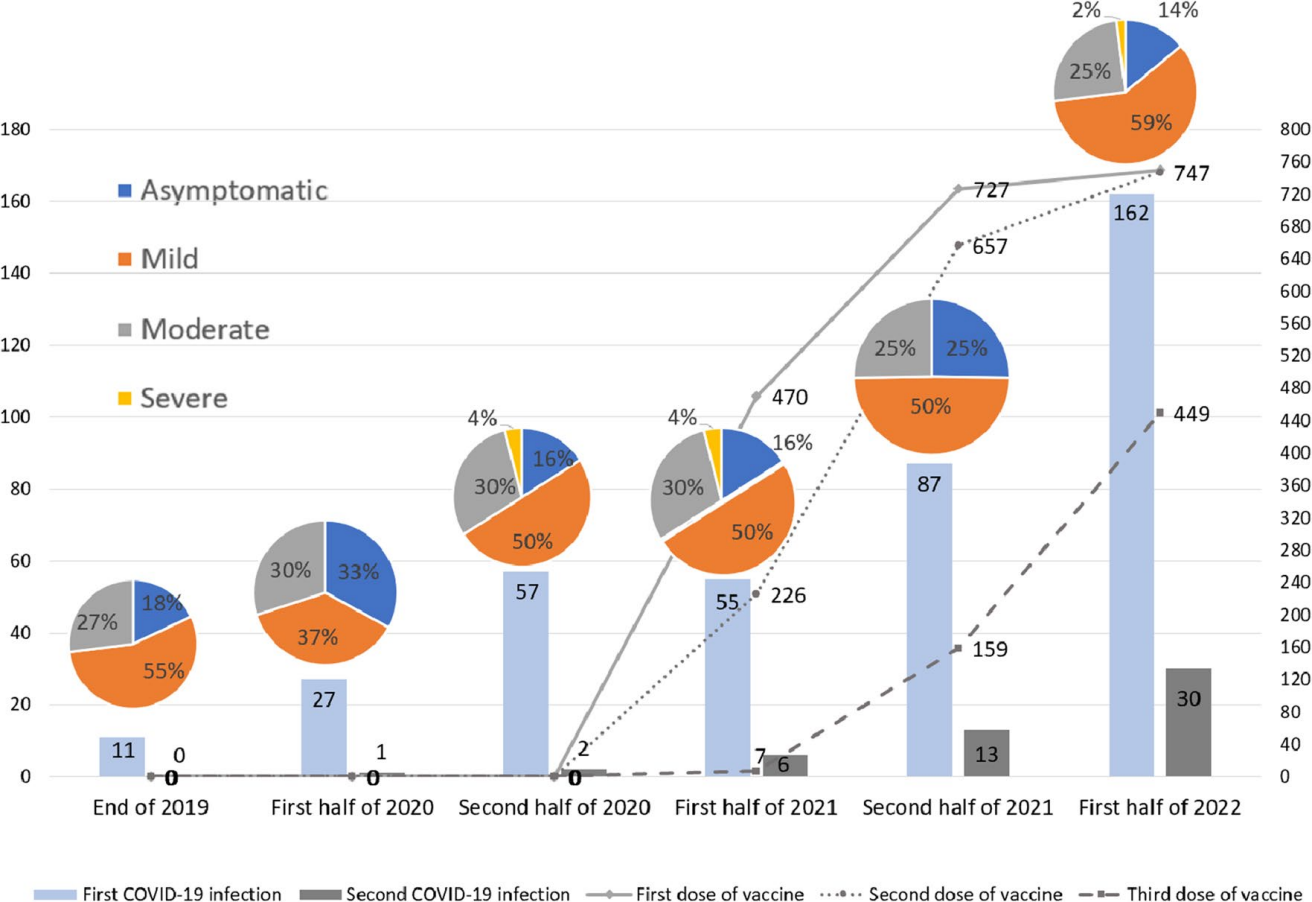
SARS-CoV-2 infection incidence and vaccination status over time. There was no significant difference in symptom severity frequencies between half-year periods.

Altogether 398 (49%) of the athletes had a SARS-CoV-2 infection at least once since the end of 2019. An additional 13% (n = 52, 6.4% of the total cohort) contracted SARS-CoV-2 twice (Fig. 1). The majority (68%) tested positive after developing symptoms suggestive of the infection. SARS-CoV-2 infection was detected during a routine screening in 24% of the cases, while 8% of the participants had a positive test during competition screening. Open water swimmers were the least (28%), while water polo (67%) and artistic swimming (61%) were the most likely to acquire the infection (p < 0.0001). A logistic regression model based on biological plausibility to predict SARS-CoV-2 contraction was developed. Increasing age (OR 1.04, CI 1.01–1.06) and being a team player (OR 2.18, CI 1.58–3.00), were independent predictors of acquiring SARS-CoV-2, while sex and training volume were not (overall model performance AUC 0.639, CI 0.61–0.67, p < 0.0001). The reinfection rate was 13% (n = 52; 28 females, 27 team players).

### Clinical characteristics of SARS-CoV-2 infection and long COVID

Most athletes had mild (54%) or moderate (27%) symptoms, and 17% reported no symptoms during the infection. Of these, only 8 (2%) required hospitalisation with severe COVID-19 symptoms. Detailed symptom frequencies for every 6-month period are shown in Table 1. Most infections occurred in the first half of 2022 (40.2%); however, the severity of the disease did not differ significantly between the examined periods. On the other hand, the missed training time differed between certain symptomatic categories. For example, in athletes with a mild infection, 1–2 weeks of missed training time was the most frequent (59%) scenario, significantly differing from asymptomatic individuals. As expectedly, an absence of more than 4 weeks from training was the most prevalent finding in athletes with a severe disease course (Table 2).

**Table 1:** The first SARS-CoV-2 infection’s symptom frequency.

N = 370	The end of 2019 (n = 11)	First half of 2020 (n = 27)	Second half of 2020 (n = 56)	First half of 2021 (n = 55)	Second half of 2021 (n = 76)	First half of 2022 (n = 145)
Smell disturbance	3 (27%)	11 (41%)	20 (36%)	17 (31%)	17 (22%)	12 (8%)
Taste disturbance	2 (18%)	10 (37%)	14 (25%)	20 (36%)	20 (26%)	14 (10%)
Headache	4 (36%)	8 (30%)	28 (50%)	22 (40%)	43 (57%)	78 (54%)
Fever	5 (45%)	10 (37%)	23 (41%)	18 (33%)	43 (57%)	70 (48%)
Severe weakness	4 (36%)	7 (26%)	22 (39%)	8 (15%)	24 (32%)	44 (30%)
Palpitation	1 (9%)	2 (7%)	5 (9%)	3 (5%)	2 (3%)	10 (7%)
Eye pain	2 (18%)	7 (26%)	8 (14%)	9 (16%)	10 (13%)	14 (10%)
Cough	3 (27%)	9 (33%)	23 (41%)	17 (31%)	46 (61%)	83 (57%)
Loss of consciousness	0 (0%)	1 (4%)	2 (4%)	2 (4%)	0 (0%)	2 (1%)
Chest pain	1 (9%)	5 (19%)	11 (20%)	7 (13%)	15 (20%)	16 (11%)
Shortness of breath	2 (18%)	5 (19%)	12 (21%)	10 (18%)	17 (22%)	28 (19%)

**Table 2:** Characteristics of the first COVID infection in terms of symptom severity.

	Asymptomatic (n = 66)	Mild (n = 216)	Moderate (n = 108)	Severe (n = 8)	P
Age (years, IQR)	23 (18–28)	23 (19–27)	22 (19–25.5)	22 (17–23)	Ns
Training volume (hours/week, IQR)	24 (20–30)	23 (20–27)	21 (17–27)	20 (20–21)	
Training history (years, IQR)	15 (12–20)	15 (11–19)	15 (12–18)	12 (6–19)	
Female, n = 253 (n, %)	42 (64%)	131 (60%)	75 (69%)	5 (62%)	
Male n = 145 (n, %)	24 (36%)	85 (40%)	33 (31%)	3 (38%)	
Long COVID (n = 40/398) (n, %)	1 (2%)^2,3^	15 (7%)^4^	22 (20%)^2,4^	2 (25%)^3^	< 0.0001
Swimming (n = 144/333) (n, %)	22 (33%)	72 (33%)	44 (41%)	6 (75%)	Ns
Open water swimming (n = 17/60), (n, %)	5 (8%)	9 (4%)	3 (3%)	0 (0%)	
Water polo (n = 89/133) (n, %)	17 (26%)	45 (21%)	26 (24%)	1 (12.5%)	
Diving (n = 50/124) (n, %)	8 (12%)	33 (15%)	9 (8%)	0 (0%)	
Artistic swimming (n = 98/162), (n, %)	14 (21%)	57 (27%)	26 (24%)	1 (12.5%)	
No days missed (n = 20) (n, %)	7 (11%)^2^	11 (5%)	2 (2%)^2^	0 (0%)	0.0013
1–3 days missed (n = 25) (n, %)	7 (11%)	9 (4%)	9 (8%)	0 (0%)	
4–6 days missed (n = 88) (n, %)	16 (24%)	53 (25%)	19 (18%)	0 (0%)	
1–2 weeks missed (n = 211) (n, %)	26 (39%)^1^	124 (57%)^1^	57 (53%)	4 (40%)	
3–4 weeks missed (n = 36) (n, %)	8 (12%)	15 (7%)	11 (10%)	2 (20%)	
> 4 weeks missed (n = 18) (n, %)	2 (3%)	4 (2%)^5^	10 (9%)^6^	2 (20%)^5,6^	

Percentage calculations are made in the context of the total number of the respective symptomatic categories for demographics, long COVID frequency, sports category and missed training calculations.

The number of positive cases per sports type is marked in brackets.

^1^p < 0.05 between asymptomatic and mildly symptomatic categories.

^2^p < 0.05 between asymptomatic and moderate categories.

^3^p < 0.05 between asymptomatic and severe categories.

^4^p < 0.05 between mildly symptomatic and moderately symptomatic categories.

^5^p < 0.05 between mildly symptomatic and severe categories.

^6^p < 0.05 between moderately symptomatic and severe categories (Chi-squared and Fisher’s exact test was used as appropriate).

Long COVID symptoms were present in 10% (40/398, 25 female) of the cases after the first infection episode (Table 2). The most common long COVID symptoms were fatigue (65%), shortness of breath (48%), cough (35%) and problems with focus and concentration (28%). The median symptom count was 1 (IQR 0–3). The missed training time category median fell into the 1–2-week interval (category 3).

A multivariate logistic regression model was built (AUC 0.76, CI 0.72–0.80, p < 0.0001) to predict long COVID occurrence. Only the symptom count during the acute, symptomatic phase of infection was a reliable, independent predictor (OR 1.4, CI 1.18–1.7), whereas age, sex and severity score did not play a significant role.

### Reinfection

Regarding reinfection (n = 52), 46% of the cases were asymptomatic, 37% showed mild symptoms, 13% had moderate, and 4% had severe symptoms. Two athletes (4%) had persisting symptoms beyond 4 weeks after the second infection. Most (65%) of the athletes missed a maximum of 6 training days after the reinfection. Most reinfections occurred during the second half of 2021 (25%) and the first half of 2022 (58%).

The second infection’s disease course was generally milder than the first infection. There was a significant difference between the symptom count and severity of the illness in those with two infections. The median symptom count was 3 (IQR 1–4) in the first and 1 (IQR 0–3) during the second infection (p < 0.0001). The median symptom severity category was “2” (mild symptoms) for both infection episodes, but interquartile ranges differed with 2–3 (mild-moderate) in the first and 1–2 (asymptomatic-mild) in the second infection (p = 0.0001). The missed training time [median category 4 (IQR 3–4) vs 3 (IQR 2–4)] intervals were shorter during the second infection than during the first infection (p = 0.0001).

### Vaccination

Ninety-four per cent of the athletes (n = 769) received at least one dose of a COVID-19 vaccine, 92% received a second dose, and 55% a third dose. Detailed vaccination data are presented in Fig. 1 and Table 3. The scores regarding vaccination experience did not differ between the subsequent rounds of shots. However, when comparing mRNA and vector vaccines, upon receiving the first shot, athletes reported slightly worse experience scores with vector vaccines than with mRNA vaccines (6 vs 8 points, p < 0.0001). During the second (9 vs 8, non-significant) and third (9 vs 8, non-significant) rounds of vaccination, experience scores were comparable.

**Table 3:** Vaccination status and side effects.

Vaccination		First dose n, (%)	Second dose n, (%)	Third dose n, (%)
Type of vaccine	Comirnaty	511 (66.4%)	538 (71.9%)	345 (76.5%)
	Spikevax	79 (10.3%)	94 (12.6%)	64 (14.2%)
	Vaxzevria	64 (8.3%)	52 (7.0%)	30 (6.6%)
	Jcovden	57 (7.4%)	15 (2%)	5 (1.1%)
	BBIBP-CorV	33 (4.3%)	33 (4.4%)	5 (1.1%)
	Gam-COVID-Vac	23 (3.0%)	15 (2.0%)	1 (0.2%)
	Nuvaoxivid	2 (0.3%)	1 (0.1%)	1 (0.2%)
Side effects of the vaccine	Yes	479 (62.2%)	398 (53.2%)	250 (55.4%)
	Local pain	319 (66.6%)	263 (66.1%)	178 (71.2%)
	Muscle or joint pain	194 (40.5%)	144 (38.0%)	94 (37.6%)
	Fever	163 (34.0%)	118 (29.6%)	73 (29.2%)
	Fatigue	148 (30.9%)	110 (27.6%)	73 (29.2%)
	Headache	145 (30.3%)	115 (30.3%)	81 (32.4%)
	Sleepiness	132 (27.6%)	101 (25.4%)	59 (23.6%)
	Swelling of lymph nodes	40 (8.4%)	45 (11.3%)	32 (12.8%)
	Palpitation	14 (2.9%)	13 (3.3%)	7 (2.8%)
	Chest pain	9 (1.9%)	8 (2.0%)	4 (1.6%)
	Vomiting	6 (1.3%)	3 (0.8%)	0 (0.0%)
Length of side effects	Unknown	0	4	2
	Less than 24 h	217	182	103
	1–3 days	234	183	122
	4–6 days	12	19	15
	1–2 weeks	7	4	6
	3–4 weeks	4	2	0
	More than 4 weeks	5	4	2
Experience of vaccination (IQR)		8 (6–10)	8 (6–10)	8 (6–10)

A higher score marks a better experience.

### Psychological aspects

The athletes provided a median of 6 (IQR 4–7) points for the overall well-being score during the pandemic. The median point for mood changes was 6 (IQR 4–8), and reduced physical performance was 5 (IQR 3–7). The severity of (the first) infection weakly correlated with the overall experience scores (rho: 0.146, p = 0.0042). Mood changes showed a strong, and subjective performance drop showed a moderate correlation with the overall experience scores (rho: 0.617, p < 0.0001 and rho: 0.466, p < 0.0001, respectively). Female athletes reported slightly worse mood change scores than male athletes (median 7 vs 6, p = 0.0002). Regarding psychological support, 19% reported a need and subsequently received psychotherapy, while 17% required assistance but could not access psychological support. However, 14% of the athletes were provided support despite not requiring it. The remaining half of the study cohort did not need or receive any help.

## Discussion

In this paper, we present the results of a comprehensive COVID-19 survey of aquatic athletes who participated in the FINA 19th World Championship, which was the first world aquatic championship after major COVID-19 waves subsided. The principal findings of this paper include a high SARS-CoV-2 infection rate in athletes, with a higher incidence in team sports. The symptoms were mostly mild; however, long COVID syndrome was detected in 10% of the athletes. High symptom counts predicted a higher burden of long COVID syndrome. The second SARS-CoV-2 infection was mostly reported to be milder, with fewer symptoms and fewer missed training days than the first. The vaccination rate among this athlete population was high (92%), predominantly reporting mild post-vaccination symptoms. Mood changes and subjective drop in performance were important in the athletes’ overall pandemic experience.

### Infection rates and clinical characteristics

The COVID-19 disease and its sequelae have affected all levels of society, including the field of professional sports. The course of infection, combined with quarantine and confinement requirements, took a toll on the quality of life of athletes during the period of the pandemic. Reduced physical activity, impaired sleep quality, altered daily activity, increased anxiety, diminished cardiorespiratory fitness, and less desirable nutritional habits were among the reported consequences^26–30^. The infection affected 8–10% of the overall population, which proved more frequent in athletes^31^. One of the largest longitudinal studies from 2020, which summarised the results of almost 10,000 athletes from 13 universities, and found a SARS-CoV-2 infection rate of 30.4%, with a 2.3% prevalence of myocarditis^32^. In this study, the overall COVID-19 infection rate was 49.4%, with 3% of severe cases requiring hospitalisation.

The higher perceived infection rate may have been due to an extended observational period and more rigorous screening of athletes. Vigorous training may also impair immune function rendering athletes more susceptible to infections with post-exercise immunosuppression through an altered helper T-cell response and elevated stress hormone levels^33,34^. This has distinct application to a cohort of elite aquatic athletes preparing for world championships.

We concluded that close physical contact is one key factor driving the increased SARS-CoV-2 infection rates, particularly among athletes in team sports. Indeed, in this study, the occurrence of infection was the highest among water polo players and artistic swimmers while the lowest among open water swimmers. This aligns with reports of higher transmission of SARS-CoV-2 infection in team sports^35,36^.

In a Swiss COVID-19 survey assessing the prevalence and symptoms of the infection in elite athletes, the disease prevalence was less than 20%, with a male predominance^35^. Interestingly, in our cohort, female athletes had higher rates of SARS-CoV-2 transmission, but symptom severity did not differ between the sexes. Other authors suggest that young female athletes have a lower risk of severe symptoms and remain dominantly asymptomatic^37,38^.

Similarly, most studies have reported mild to moderate COVID-19 symptoms in athletes^32,35,39–41^. In our findings, the main symptoms were headache, fever and cough. Different SARS-CoV-2 variants have been linked to distinct symptom profiles and diseases. In a prospective registry of collegiate athletes, the most common symptoms detected between September and December 2020 were loss of taste or smell, headache, muscle pain, cough, fatigue and fever, similar to our data gained about the second half of the year 2020^39^.

However, the exact missed training time interval is scarcely reported in athletes and may be dependent on several factors. In our findings, most athletes missed 1–2 weeks of training which might involve the role of quarantine, symptom severity, and closure of training sites, among others. According to Krzywanski et al.’s findings, COVID-19’s impact has been higher than other respiratory tract infections regarding missed training. The surveyed elite Polish athletes (n = 1073) reported that 12–13% lost training days due to COVID-19, which is expressly lower than what the multinational aquatic athletes showed^42^. Schwellnus et al. associated symptom clustering with different return-to-play times and compared COVID-19 with other acute respiratory illnesses. They found that return-to-play after COVID-19 was significantly longer than in the other group (IQRs 16–40 vs 7–22 days) and, generally, symptom cluster is associated with prolonged missed training times with excess fatigue in particular^43^.

### Long COVID

Our study detected long COVID syndrome in 10% of the athletes. A systematic review and meta-analysis of 43 studies covering 11,518 athletes reported a long COVID syndrome rate of 8.3%^7^. A study including 4186 post-COVID patients assessed the prevalence of long COVID syndrome in the average population and found a slightly higher incidence (13.3%) of persistent (> 4 weeks) symptoms^8^. Our previous findings in a prospective cohort of 322 athletes showed a long COVID occurrence of 8%, highlighting differences between age groups, where adult and master athletes were more likely to develop long-standing symptoms^5^. We previously showed that increasing age and a worse symptom severity score predicted long COVID occurrence. Nevertheless, in this dataset, including predominantly young adults, we found that only acute symptom count was an independent predictor of developing long COVID.

### Reinfection

The SARS-CoV-2 reinfection of athletes has not been extensively described in the literature, with case reports and low case number studies available^44,45^. Good et al. reported that the reinfection rate was as low as 0.8% in a pre-omicron (Delta wave) student-athlete cohort. In our study, the reinfection rate was 13%, with fewer and milder symptoms and fewer missed training days than the first infection. This phenomenon may be related to the different SARS-CoV-2 variants^22,46,47^. Another explanation might be that athletes encountered the second infection with an already primed immune system either through prior infection or vaccination. Another insight might be that vaccine roll-out helped mitigate the disease severity. In addition, our relatively high reinfection rate may be explained by the fact that most SARS-CoV-2 infections occurred in the first half of 2022 during the Omicron variant outbreak. Furthermore, elite athletes usually have strict medical control due to the pre-competition screenings and regular health check-ups; thus, it is more likely that mild or asymptomatic (re)infections are recognized. In contrast, the reinfection rate in the non-athletic population was relatively low until the Omicron era^37,48^.

### Vaccination

Almost all (94%) of our participants had been administered at least one dose of a COVID-19 vaccine, and 92% received the second shot as well. With a high vaccination rate, the athletes reported a relatively low burden of adverse side effects and only a few major ones, similar to the general population^21^. The duration of side effects was generally short, lasting no more than three days. An infographic paper by Rankin et al. found that the vaccination in athletes was generally well tolerated, the majority of the side effects were mild, and the missed training period was as low as 4 days^24,49^. Oudjedi et al. found that 55% of the surveyed Algerian athletes (n = 273) reported side effects after COVID-19 vaccination, of which fever and local pain were the most prevalent. These findings generally align with our data. Nonetheless, for athletes with the dominant use of the upper extremities, the timing of the vaccine administration may be decisive due to the possible local side effects^24^.

In spite of the increasing SARS-CoV-2 vaccination rate, the number of infected cases also surged, which may reflect the lower efficacy of the vaccines against the newer Omicron variants as described in the literature^47,50,51^.

### Psychological aspects

The COVID-19 pandemic has brought uncertainty to the lives of athletes and non-athletes, leading to stress, anxiety, depression and other mental disorders^52^. A study examining 310 athletes from different continents and diverse sports disciplines found that maladaptive perfectionism was related to numerous mental health indicators. They also concluded that competitive athletes showed signs of a negative emotional state during the pandemic. Even so, the anxiety, stress, and depressive symptoms were less prevalent in athletes with proper coping strategies^53^. Based on our results, mood changes and subjective performance drop correlated closely with the overall experience during the pandemic; 36% of the athletes reported needing psychological support. Female athletes reported slightly worse mood change scores compared to male athletes. Several studies found similar data regarding the post-COVID mental health of female athletes^20,38,54,55^. Notably, half of the athletes questioned did not need psychological support. However, it is not apparent whether this is reflective of adequate coping skills—such as cognitive restructuring and emotional calm—or not. Also, 17% of the surveyed athletes were unable to obtain psychological support despite their need, reflecting the importance of providing appropriate psychological help in the context of sport.

### Limitations

This self-reported, retrospective study is based on a monolingual (English) survey, and the language barrier may have impacted the answers of non-native English speakers. In addition, we do not have any information about the type and methods of SARS-CoV-2 testing, nor could we confirm the validity of other reported health-related variables. Consequently, the potential lack of accuracy and incompleteness of the answer sheets may have slightly influenced the data quality. In addition, the exact date of any positive tests could not be verified through institutional medical sources.

## Conclusion

In this multinational cohort study of elite aquatic athletes, despite a high SARS-CoV-2 infection rate, the majority experienced only mild to moderate symptoms, while athletes with more severe disease courses had a higher incidence of long COVID syndrome. Team sports participants presented with a higher infection transmission rate compared to individual athletes, likely due to close bodily contact. The 13% reinfection rate aligns with previously published research findings. Promisingly, nearly all athletes had received at least two doses of the COVID-19 vaccine, indicating a broad vaccination acceptance in aquatic athletes worldwide, with short-term and mild side effects. Notably, psychological aspects significantly influenced athletes’ perceptions of the pandemic. Overall, this study suggests that aquatic athletes have coped well with the physical and moderately well with the psychological consequences of the COVID-19 pandemic. Nevertheless, post-pandemic follow-up will be crucial to assess the complex effects on athletes’ long-term physical and mental health. The findings of the study can offer guidance to sports organizations and health authorities in designing targeted strategies for pandemic preparedness tailored to elite aquatic athletes. The higher infection transmission rates in team sports underscore the importance of implementing specific safety measures during training and competing to minimize viral spread. In addition, the high vaccination acceptance rate among athletes highlights the importance of promoting vaccination in sports communities to ensure a safer and more resilient sports milieu during potential future health crises. Furthermore, the acknowledgement of the impact of psychological aspects on athletes’ pandemic experience emphasizes the need for continued support and psychological interventions to safeguard athletes’ mental well-being during challenging times. These pragmatic implications can enhance the overall health of elite aquatic athletes in the face of future pandemics or health challenges.

### Supplementary Information

Supplementary Information.

## Data Availability

All data are available upon reasonable request from the corresponding author (Hajnalka Vágó M.D., PhD, vago.hajnalka@semmelweis.hu) and not made available by default due to possible compromise of individual privacy.
