# Supplementary material for: Retrospective study of COVID-19 experiences in elite multinational aquatic athletes
Source: Sci Rep. 2023 Aug 26;13:13978. doi: 10.1038/s41598-023-40821-2 (PMC10460449; doi:10.1038/s41598-023-40821-2)
Supplement: Supplementary file 1 — Supplementary Information. [file 41598_2023_40821_MOESM1_ESM.pdf]

COVID-19 questionnaire - FINA 2022

Dear Athlete,

We are happy to welcome you in Budapest for FINA 2022!
We invite you to fill in this questionnaire about your experience with COVID-19 and COVID vaccination. We also ask questions about your sports activity.
Your answers will be used anonymously in scientific research conducted by FINA and the Heart and Vascular Center of Semmelweis University, Budapest.

Filling in the form takes about 5-10 minutes. Some of the questions are not mandatory and only apply to certain sports types.

Thank you for your time and answers,
On behalf of the Medical Staff

*Required

1. Your sex *

Mark only one oval.

- Female
- Male
- Prefer not to say

2. Nationality *

____

3. Age *

____

4. Body weight (kg or lbs - please mark) *

____

5. Height (cm or ft + in - please mark) *

____

6. Your e-mail address (optional)

____

Sports activity

7. On average, how many HOURS do you train per week? *

____

8. How many YEARS have you been doing sports? *

____

9. What is your (primary) sport type? *

Mark only one oval.

- ( ) Swimming *Skip to question 10*
- ( ) Waterpolo *Skip to question 12*
- ( ) Artistic swimming *Skip to question 10*
- ( ) Diving *Skip to question 12*
- ( ) High diving *Skip to question 12*
- ( ) Open water swimming *Skip to question 10*

This section applies to you only if you are a swimmer

10. Which swim distance do you compete in?

Tick all that apply.

- [ ] 50 m
- [ ] 100 m
- [ ] 200 m
- [ ] 400 m
- [ ] 800 m
- [ ] 1500 m
- [ ] Open water (5 km)
- [ ] Open water (10 km)
- [ ] Open water (25 km)

11. Which swimming stroke do you compete in?

Tick all that apply.

- [ ] butterfly
- [ ] backstroke
- [ ] breaststroke
- [ ] freestyle
- [ ] medley
- [ ] open water

COVID-19 and testing

12. Did you ever have a positive test for COVID? *

Mark only one oval.

- ( ) Yes *Skip to question 13*
- ( ) No *Skip to question 31*

Fill in this section if you have been infected with COVID at least once

13. When was your 1st positive PCR/COVID rapid test taken, if any? If you don't know the exact date, please move on to the next question

Example: 7 January 2019

14. When was your 1st positive PCR/COVID test taken? *

Mark only one oval.

- Second half of 2019
- First half of 2020
- Second half of 2020
- First half of 2021
- Second half of 2021
- First half of 2022

15. Why did they perform COVID testing on you when it was positive? *

Mark only one oval.

- I had symptoms
- Routine screening
- It was mandatory for competition

16. How long did the symptoms last? *

Mark only one oval.

- I had no symptoms
- < 1 day
- 1-3 days
- 4-6 days
- 1-2 weeks
- 3-4 weeks
- More than 4 weeks

17. For how long did you miss training due to the COVID infection? *

Mark only one oval.

- No days missed
- 1-3 days
- 4-6 days
- 1-2 weeks
- 3-4 weeks
- More than 4 weeks

18. During the illness did you have: *

Tick all that apply.

- I had no symptoms
- Chest pain
- Fever
- Taste disturbance
- Smell disturbance
- Palpitation (fast heart rate)
- Severe weakness
- Loss of consciousness
- Cough
- Shortness of breath
- Headache
- Eye pain
- Sensation of burnt skin
- Other: ____________________

19. Did you need hospital care as a result of the illness? *

Mark only one oval.

- Yes
- No

20. Did you have any long-standing symptoms for more than 4 weeks? (long COVID) *

Mark only one oval.

- ( ) Yes *Skip to question 21*
- ( ) No *Skip to question 22*

Long COVID symptoms

21. Please choose your long COVID symptoms:

Tick all that apply.

- [ ] Fatigue
- [ ] Headache
- [ ] Palpitation (fast heart rate)
- [ ] Cough
- [ ] Loss of smell and/or taste
- [ ] Dizziness
- [ ] Problem with focus and concentration
- [ ] Mood changes
- [ ] Depression
- [ ] Weakness (physical)
- [ ] Shortness of breath
- [ ] Other: ____________________

Second COVID infection

22. Did you have a second COVID infection? *

Mark only one oval.

- ( ) Yes *Skip to question 23*
- ( ) No *Skip to question 31*

Fill in this section if you have been infected with COVID twice (2x). This section applies to your 2nd infection

23. When was your 2nd postitive PCR/COVID rapid test taken? If you don't know the exact date, please move on to the next question

____

Example: 7 January 2019

24. When was your 2nd positive PCR/COVID test taken? *

Mark only one oval.

- ⬭ First half of 2020
- ⬭ Second half of 2020
- ⬭ First half of 2021
- ⬭ Second half of 2021
- ⬭ First half of 2022

25. How long did the symptoms last? *

Mark only one oval.

- ⬭ I had no symptoms
- ⬭ < 1 day
- ⬭ 1-3 days
- ⬭ 4-6 days
- ⬭ 1-2 weeks
- ⬭ 3-4 weeks
- ⬭ More than 4 weeks

26. For how long did you miss training due to the second COVID infection? *

Mark only one oval.

- ( ) No days left out
- ( ) 1-3 days
- ( ) 4-6 days
- ( ) 1-2 weeks
- ( ) 3-4 weeks
- ( ) More than 4 weeks

27. During the illness did you have: *

Tick all that apply.

- [ ] I had no symptoms
- [ ] Chest pain
- [ ] Fever
- [ ] Taste disturbance
- [ ] Smell disturbance
- [ ] Palpitation (fast heart rate)
- [ ] Severe weakness
- [ ] Loss of consciousness
- [ ] Cough
- [ ] Shortness of breath
- [ ] Headache
- [ ] Eye pain
- [ ] Sensation of burnt skin
- [ ] Other: ____________

28. Did you need hospital care as a result of the illness? *

Mark only one oval.

- ( ) Yes
- ( ) No

29. Did you have any long-standing symptoms for more than 4 weeks? (long COVID) *

Mark only one oval.

- Yes *Skip to question 30*
- No *Skip to question 31*

Long COVID symptoms (2nd infection)

30. If yes, please choose your long COVID symptoms:

Tick all that apply.

- [ ] Fatigue
- [ ] Headache
- [ ] Palpitation (fast heart rate)
- [ ] Cough
- [ ] Loss of smell and/or taste
- [ ] Dizziness
- [ ] Problem with focus and concentration
- [ ] Mood changes
- [ ] Depression
- [ ] Weakness (physical)
- [ ] Shortness of breath
- [ ] Other: ____________________

COVID-19 vaccination

31. Did you receive any COVID vaccination? *

Mark only one oval.

- Yes *Skip to question 32*
- No *Skip to question 55*

COVID-19 Vaccination (1st shot)

32. Date of the 1st dose of COVID vaccination? If you don't know the exact date, please move on to the next question

____

Example: 7 January 2019

33. Date of the 1st dose of COVID vaccination:

Mark only one oval.

- ( ) The end of 2020
- ( ) First half of 2021
- ( ) Second half of 2021
- ( ) First half of 2022

34. What kind of COVID vaccination did you get (1st shot)? *

Mark only one oval.

- ( ) Pfizer
- ( ) AstraZeneca
- ( ) Moderna
- ( ) Sputnik
- ( ) Janssen
- ( ) Sinopharm
- ( ) Novavax

35. Did you have any side effect of the vaccination? *

Mark only one oval.

- ( ) Yes
- ( ) No *Skip to question 39*

36. What kind of side effect(s) did you experience?

Tick all that apply.

- [ ] Local pain at the injection site
- [ ] Sleepiness
- [ ] Fever
- [ ] Palpitation/fast heart rate
- [ ] Swelling of lymph nodes (e.g. armpit)
- [ ] Headache
- [ ] Weakness/fatigue
- [ ] Muscle pain
- [ ] Joint pain
- [ ] Vomiting
- [ ] Chest pain
- [ ] Other: ______________________

37. How long did the symptoms last?

Mark only one oval.

- ( ) Less than 24 hours
- ( ) 1-3 days
- ( ) 4-6 days
- ( ) 1-2 weeks
- ( ) 2-3 weeks
- ( ) More than 3 weeks

38. How was your experience with your 1st shot? *

Mark only one oval.

Horrible experience

1 ⬭
2 ⬭
3 ⬭
4 ⬭
5 ⬭
6 ⬭
7 ⬭
8 ⬭
9 ⬭
10 ⬭

Did not make any difference

Skip to question 39

COVID-19 vaccination (2nd shot)

39. Did you receive a 2nd dose of COVID vaccination? *

Mark only one oval.

- Yes *Skip to question 40*
- No *Skip to question 55*

COVID-19 vaccination (2nd shot)

40. Date of the 2nd dose of COVID vaccination? If you don't know the exact date, please move on to the next question

Example: 7 January 2019

41. Date of the 2nd dose of COVID vaccionation: *

Mark only one oval.

- The end of 2020
- First half of 2021
- Second half of 2021
- First half of 2022

42. What kind of COVID vaccination did you get? *

Mark only one oval.

- Pfizer
- AstraZeneca
- Moderna
- Sputnik
- Janssen
- Sinopharm
- Novavax

43. Did you have any side effect of the vaccination? *

Mark only one oval.

- ( ) Yes
- ( ) No

44. What kind of side effect(s) did you experience?

Tick all that apply.

- [ ] Local pain at the injection site
- [ ] Sleepiness
- [ ] Fever
- [ ] Palpitation/fast heart rate
- [ ] Swelling of lymph nodes (e.g. armpit)
- [ ] Headache
- [ ] Weakness/fatigue
- [ ] Muscle pain
- [ ] Joint pain
- [ ] Vomiting
- [ ] Chest pain
- [ ] Other: ____________

45. How long did the symptoms last?

Mark only one oval.

- ( ) Less than 24 hours
- ( ) 1-3 days
- ( ) 4-6 days
- ( ) 1-2 weeks
- ( ) 2-3 weeks
- ( ) More than 3 weeks

46. How was your experience with your 2nd shot? *

Mark only one oval.

Horrible experience

1 ◯
2 ◯
3 ◯
4 ◯
5 ◯
6 ◯
7 ◯
8 ◯
9 ◯
10 ◯

Did not make any difference

Skip to question 47

COVID-19 vaccination (3rd shot)

47. Did you receive the 3rd dose of COVID vaccination? *

Mark only one oval.

- ( ) Yes *Skip to question 48*
- ( ) No *Skip to question 55*

COVID-19 vaccination (3rd shot)

48. Date of the 3rd dose of COVID vaccination? If you don't know the exact date, please move on to the next question

____

Example: 7 January 2019

49. Date of the 3rd dose of COVID vaccionation: *

Mark only one oval.

- ( ) First half of 2021
- ( ) Second half of 2021
- ( ) First half of 2022

50. What kind of vaccination did you get for the 3rd time? *

Mark only one oval.

- ( ) Pfizer
- ( ) AstraZeneca
- ( ) Moderna
- ( ) Sputnik
- ( ) Janssen
- ( ) Sinopharm
- ( ) Novavax

51. Did you have any side effect of the vaccination? *

Mark only one oval.

- Yes
- No

52. What kind of side effect(s) did you experience?

Tick all that apply.

- Local pain at the injection site
- Sleepiness
- Fever
- Palpitation/fast heart rate
- Swelling of lymph nodes (e.g. armpit)
- Headache
- Weakness/fatigue
- Muscle pain
- Joint pain
- Chest pain
- Other: ____________________

53. How long did the symptoms last?

Mark only one oval.

- Less than 24 hours
- 1-3 days
- 4-6 days
- 1-2 weeks
- 2-3 weeks
- More than 3 weeks

54. How was your experience with your 3rd shot? *

Mark only one oval.

	Horrible experience
1	◯
2	◯
3	◯
4	◯
5	◯
6	◯
7	◯
8	◯
9	◯
10	◯
	Did not make any difference

Mental and psychological aspects of COVID-19

55. How has the pandemic affected you so far? *

Mark only one oval.

It did not make any difference to me

1 ◯
2 ◯
3 ◯
4 ◯
5 ◯
6 ◯
7 ◯
8 ◯
9 ◯
10 ◯

Absolutely terrible

56. Did you experience mood changes through the pandemic? *

Mark only one oval.

Not at all

1 ◯
2 ◯
3 ◯
4 ◯
5 ◯
6 ◯
7 ◯
8 ◯
9 ◯
10 ◯

Very much

57. How much did your performance drop (according to your feelings) after restarting to compete compared to before the pandemic? *

Mark only one oval.

Not at all

1 ◯
2 ◯
3 ◯
4 ◯
5 ◯
6 ◯
7 ◯
8 ◯
9 ◯
10 ◯

Very bad

58. Psychological support during the pandemic *

Mark only one oval.

- I needed and I got psychological support
- I needed but I did not get psychological support
- I did not need it, but I got psychological support
- I did not need it and I did not get psychological support

59. Is there anything you would like to add about your experience with the COVID-19 pandemic?

____

____

____

____

____

Consent to anonymous data usage

60. Do you give your consent to Semmelweis University and FINA to the fully anonymous usage of the data provided through this questionnaire for scientific purposes? *

Mark only one oval.

- Yes
- No (if you choose this option, we cannot use your answers)

This content is neither created nor endorsed by Google.

Google Forms
